# Case Report: A rare stop-gained *MYZAP* mutation is associated with atrioventricular septal defects in an Arabian family

**DOI:** 10.3389/fcvm.2026.1866593

**Published:** 2026-07-20

**Authors:** Zaher Zaher, Gaser Abdelmohsen, Saud Bahaidarah, Faris Baamer, Angham Abdulrhman Abdulkareem, Abdulmajeed F. Alrefaei, Muhammad Imran Naseer, Muhammad Abu-Elmagd

**Affiliations:** 1 Paediatric Cardiology Division, Department of Paediatrics, King Abdulaziz University, Jeddah, Saudi Arabia; 2 Paediatric Cardiology Division, Department of Paediatrics, Kasr Al Ainy School of Medicine, Cairo University, Cairo, Egypt; 3 King Abdullah Medical Complex, Ministry of Health, Jeddah, Saudi Arabia; 4 Department of Biochemistry, Faculty of Sciences, King Abdulaziz University, Jeddah, Saudi Arabia; 5 Institute of Genomic Medicine Sciences (IGMS), King Abdulaziz University, Jeddah, Saudi Arabia; 6 Department of Biology, Jamoum University College, Umm Al-Qura University, Makkah, Saudi Arabia

**Keywords:** atrioventricular septal defects (AVSD), *MYZAP*, Saudi Arabia, stop-gain, whole exome sequencing (WES)

## Abstract

**Background:**

Atrioventricular septal defects (AVSDs) represent a diverse group of congenital cardiac malformations arising from abnormal development of the endocardial cushions. Although several transcription factors have been implicated in septation, the contributions of structural proteins involved in cardiomyocyte integrity remain incompletely understood.

**Methods and Results:**

We investigated an Arabian family presenting with a spectrum of AVSD phenotypes, including complete AVSD, partial AVSD, and an isolated cleft of the anterior mitral leaflet. Whole-exome sequencing identified a novel homozygous stop-gain variant in the *MYZAP* gene (NM_001018100.5:c.229C > T; p.Arg77Ter) in two affected siblings. Segregation analysis confirmed heterozygous carriage in the father and absence in the unaffected mother. The variant is extremely rare in population databases and meets ACMG criteria for likely pathogenicity.

**Conclusion:**

This report expands the phenotypic spectrum associated with *MYZAP* and suggests a potential role in cardiac septation. While *MYZAP* is primarily linked to cardiomyopathy, our findings raise the possibility that it may contribute to congenital cardiac malformations, potentially through disruption of cardiomyocyte adhesion or developmental signaling pathways controling cardiogenesis. Further functional studies are required to validate this association.

## 1 Introduction

Atrioventricular septal defect (AVSD) is a spectrum of congenital cardiac anomalies that is strongly associated with trisomy 21 (Down syndrome) cases. Approximately 30%–40% of trisomy 21 cases developed AVSD, making it one of the most characteristic congenital heart defects (1, 2). The incidence of this septal defect ranges from 4 to 5 per 10,000 live births, accounting for approximately 6% of all congenital cardiac defects (3). An embryological defect in the development of the endocardial cushions during cardiac development causes the AVSD spectrum. These cushions are crucial for forming the atrial and ventricular septa and the two separate atrioventricular valves in the normal heart. Therefore, abnormal embryonic development of the endocardial cushions may lead to the induction of the AVSD spectrum (4). This spectrum includes either the complete, partial, intermediate, or the transitional forms, canal type of ventricular septal defect (VSD), and the isolated cleft of the anterior mitral valve leaflet (5, 6). The complete form has a large primum atrial septal defect (ASD), a common atrioventricular valve, and a large inlet VSD. The partial form of AVSD is primum ASD, with two separate atrioventricular valves and a cleft in the anterior mitral leaflet. The partial form of AVSD develops due to partial fusion between the superior and inferior bridging leaflets, with complete adherence of the atrioventricular valve to the crest of the ventricular septum, thus preventing interventricular shunting that leads to no VSD formation.

The intermediate form is similar to the complete form (ASD primum, large-inlet VSD), but with two atrioventricular valves. The transitional type is identical to the partial form but with a small inlet VSD. Isolated canal-type VSD and isolated cleft in the anterior mitral leaflet are also considered a spectrum of AVSD (5, 6). In the current case study, we reported on a family with a spectrum of AVSD. The father had the partial form; one daughter had the complete form, while the other daughter had an isolated cleft in the anterior mitral leaflet. None of the family members had dysmorphic features.

## 2 Subjects and methods

### 2.1 Ethical approval

This study was conducted in accordance with the Declaration of Helsinki and approved by the Biomedical Ethics Committee of King Abdulaziz University Hospital (KAUH) (Approval No.: 53-25), Jeddah, Saudi Arabia. Informed consent was obtained from all subjects involved in the study, including written informed consent to use the study data for publication.

### 2.2 Subjects

A family of Arabian origin was evaluated following referral for congenital heart disease. Clinical assessment included echocardiography and electrocardiography for all affected individuals. Cardiac catheterization was performed when clinically indicated. All affected individuals enrolled in the present study were clinically evaluated for dysmorphic features and/or phenotypic characteristics suggestive of Down syndrome. In addition, chromosomal analysis (karyotyping) was performed in the affected children to exclude underlying chromosomal abnormalities, including trisomy 21.

### 2.3 Exome sequencing and analysis

Whole-exome sequencing (WES) was performed on three individuals from the Arab family, including father and his two affected twin daughters, using the Illumina NextSeq 6000 platform with the High-Output v2 kit, generating 2 × 76 bp paired-end reads. Whole-exome sequencing achieved a mean target coverage depth of approximately 85×, with 94.6% of targeted bases covered at ≥20× depth. Although sequencing was performed using 2 × 76 bp paired-end reads on the Illumina NextSeq platform, coverage across the *MYZAP* locus, including exon 3 containing the c.229C > T variant, was adequate for reliable variant detection and was subsequently confirmed by Sanger sequencing Library preparation was carried out using standard quantitative procedures, including the use of Roche's Rapid Library Quantification System to establish an accurate standard curve and determine library concentration. Raw sequencing reads were subjected to quality control assessment and aligned to the human reference genome (GRCh37/hg19, as applicable) using the Burrows-Wheeler Aligner (BWA-MEM). Duplicate reads were marked and removed, and variant calling was performed using the Genome Analysis Toolkit (GATK) Best Practices workflow. Single nucleotide variants (SNVs) and small insertions/deletions (indels) were identified and subsequently annotated using ANNOVAR (or VEP/SnpEff) (7, 8).

Variants were filtered based on the following criteria: (i) exonic or splice-site location; (ii) minor allele frequency (MAF) <0.01 in population databases; (iii) predicted functional impact including missense, nonsense, frameshift, or splice-site variants; and (iv) consistency with the suspected inheritance pattern. Candidate variants were prioritized according to gene function, known disease associations, segregation within the family, phenotypic relevance, and ACMG/AMP guidelines. Variant annotation and filtration were performed within the Golden Helix VarSeq environment, which incorporates the American College of Medical Genetics and Genomics (ACMG) guideline-based classification. Multiple population, clinical, and functional databases, including gnomAD, 1000 Genomes, ClinVar, OMIM, dbSNP, RefSeq, and ExAC, and gene constraint metrics were used to assess allele frequency and clinical relevance. A broad set of *in silico* prediction tools (including SIFT, PolyPhen-2, GERP++, PhyloP, GeneSplicer, NNSplice, and PWM splice predictors) was applied to evaluate the potential impact of amino-acid substitutions and splice-site alterations. All variants were annotated according to HGVS nomenclature conventions using the VarSeq transcript annotation workflow, ensuring consistency with the most frequently referenced transcripts in ClinVar.

Rare and potentially pathogenic variants (MAF ≤ 0.01%), including both homozygous and heterozygous alterations, were prioritized based on computational pathogenicity predictions, genomic context, and correlation with the clinical phenotype. Variant interpretation adhered strictly to the ACMG/AMP 2015 standards. Additional computational analyses, including MutationTaster, PhastCons, SiPhy, CADD, GERP++, and other evolutionary conservation metrics, were applied to further the deleterious nature of the candidate variant and its potential association with disease causation. The identified *MYZAP* variant was further validated by Sanger sequencing in all available family members. Variant pathogenicity was assessed according to ACMG/AMP recommendations using available population, computational, segregation, and functional evidence.

### 2.4 Sanger sequencing

The Sanger sequencing was used to segregate the identified mutation in the remaining family members. For PCR and sequencing, sets of targeted primers were designed using the Primer3 program. Primer sequences were as follows: forward primer MYZP-Ex3F: 5'-ACAGTGGGCCCGTTATTACA-3' and reverse primer MYZP-E3xR: 5'-CACTTCAATGCTTCCAACTCCA-3'. Sequencing data files were obtained from the AB1 sequencing unit. The sequencing file was aligned with the reference sequence using BioEdit software. The study was carried out according to the guidelines of the National Center for Biotechnology Information (NCBI) SNP database.

## 3 Results

### 3.1 Cases presentation

**Figure 1:**
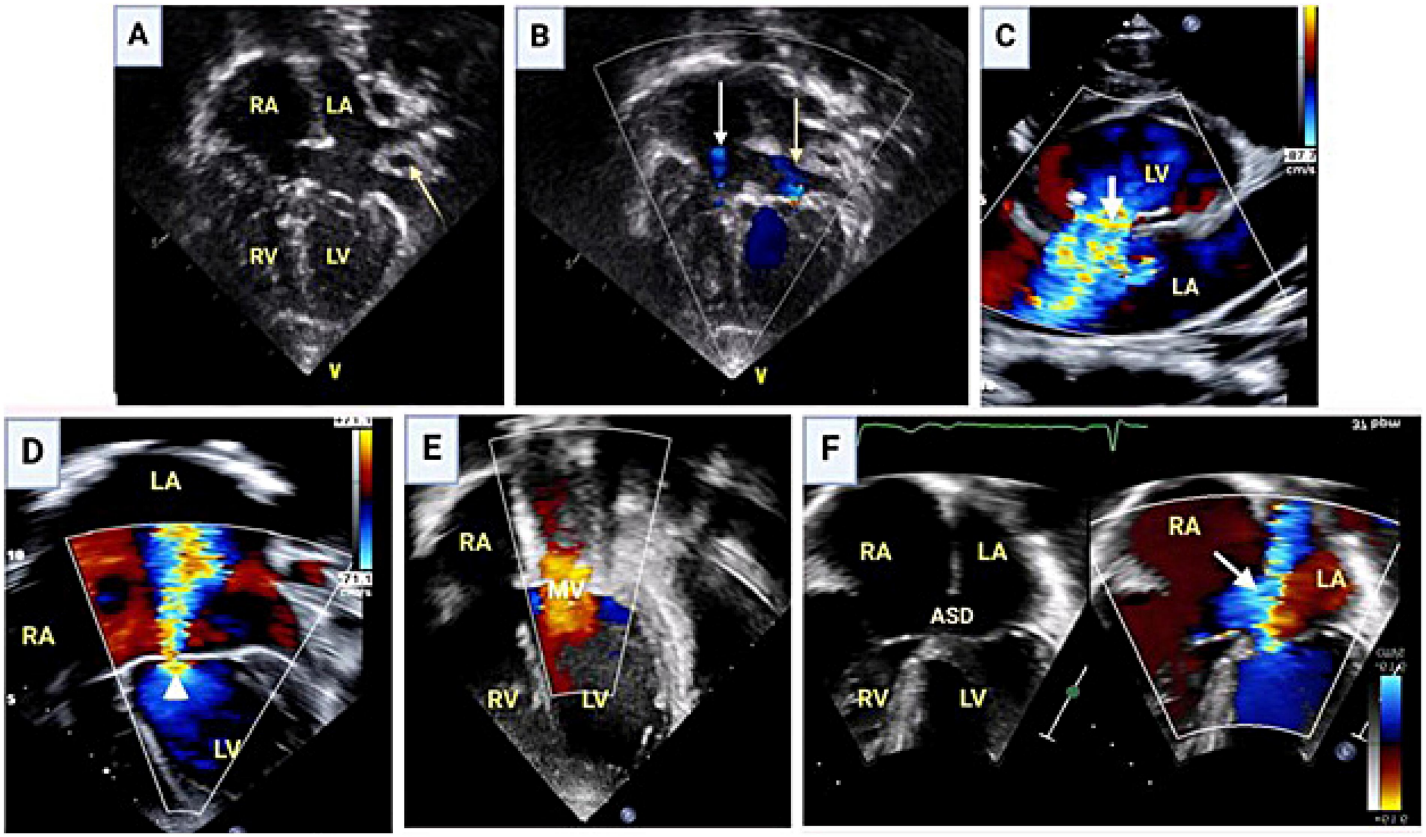
Echocardiography of the patients. **(A)** Echocardiography with the apical 4-chamber view of the first patient was done during the last follow-up, showing a dilated coronary sinus due to persistent left SVC (yellow arrow). **(B)** An apical 4-chamber view with color flow Doppler of the first patient was performed at the last follow-up and showed mild TR (white arrow) and mild MR (yellow arrow). **(C)** Preoperative modified parasternal short-axis view of the second patient showing a large cleft in the anterior mitral leaflet with severe MR (white arrow). **(D)** Preoperative apical 4-chamber view of the second patient with color flow Doppler showing MR jet passing through the body of the anterior mitral leaflet (arrowhead). **(E)** An apical 4-chamber view of the second patient after the mechanical mitral valve prosthesis, showing laminar blood flow across the mitral inflow. **(F)** Preoperative apical four-chamber view in 2D (left) and color flow Doppler (right) of the father showing ASD primum with a cleft in the anterior mitral leaflet causing mild to moderate MR (white arrow). ASD, atrial septal defect; LA, left atrium; LV, left ventricle; MV, mitral valve; MR, mitral regurgitation; RA, right atrium; RV, right ventricle; SVC, superior vena cava; TR, tricuspid regurgitation.

The first patient was a baby girl who presented at the age of 2 months with the manifestation of heart failure with dyspnea and diaphoresis on feeding and interrupted feeding. Echocardiography revealed a balanced complete AVSD with a large ASD, common atrioventricular valve, large inlet VSD, absent right superior vena cava (SVC) with persistent left SVC draining into the coronary sinus (CS), and coarctation of the aorta (COA). The patient underwent palliative surgery in the form of COA repair (resection with end-to-end anastomosis) and pulmonary artery banding. At 10 months of age, the patient underwent surgical correction. At the age of 3 years, the patient developed progressive left ventricular outflow tract obstruction (LVOTO) due to a fibromuscular ridge and underwent resection with good results. The patient is now 8 years old, doing well, and not on cardiac medication; the last echocardiography revealed mild mitral regurgitation (MR), mild tricuspid regurgitation (TR), good cardiac function, no residual ASD or VSD, and no LVOTO (Figures 1A,B). The second patient was presented to us at the age of 5 years with exercise intolerance and a wheezy chest. Echocardiography revealed a large cleft in the anterior mitral leaflet opposite the inlet septum with severe mitral regurgitation and a gigantic left atrium (Figures 1C,D). The patient underwent mitral valve repair, during which the cleft was closed, and a posterior annuloplasty was performed.

Postoperative echocardiogram revealed residual severe MR, so redo surgery was done ten days later, where mitral valve replacement using a mechanical mitral valve was carried out. After surgery, the patient had complete heart block and underwent a permanent dual-chamber epicardial pacemaker. The patient is now 8 years old and doing fine. The last echocardiogram revealed good cardiac function with a functioning mechanical mitral valve (Figure 1E); the mean inflow gradient across the mitral valve is 3 mmHg with no paravalvular leak and normal pulmonary artery pressure. The last case presented to us was the father, who was 39 years old. The patient had frequent syncopal attacks. The heart rate was 35/minute. ECG revealed a complete heart block, and echocardiogram revealed partial AVSD with large ASD primum, cleft in the anterior mitral leaflet with mild to moderate MR, and no VSD (Figure 1F). The patient underwent temporary transvenous pacing and then underwent surgical correction with a permanent dual-chamber epicardial pacemaker. The patient is now doing fine. Cardiac function in all family members was normal, and no underlying cardiomyopathy was associated. None of the affected individuals demonstrated clinical features suggestive of Down syndrome or other syndromic chromosomal disorders. Conventional karyotype analysis performed in the affected children revealed normal chromosomal findings with no evidence of trisomy 21.

### 3.2 Whole exome sequencing

**Figure 2:**
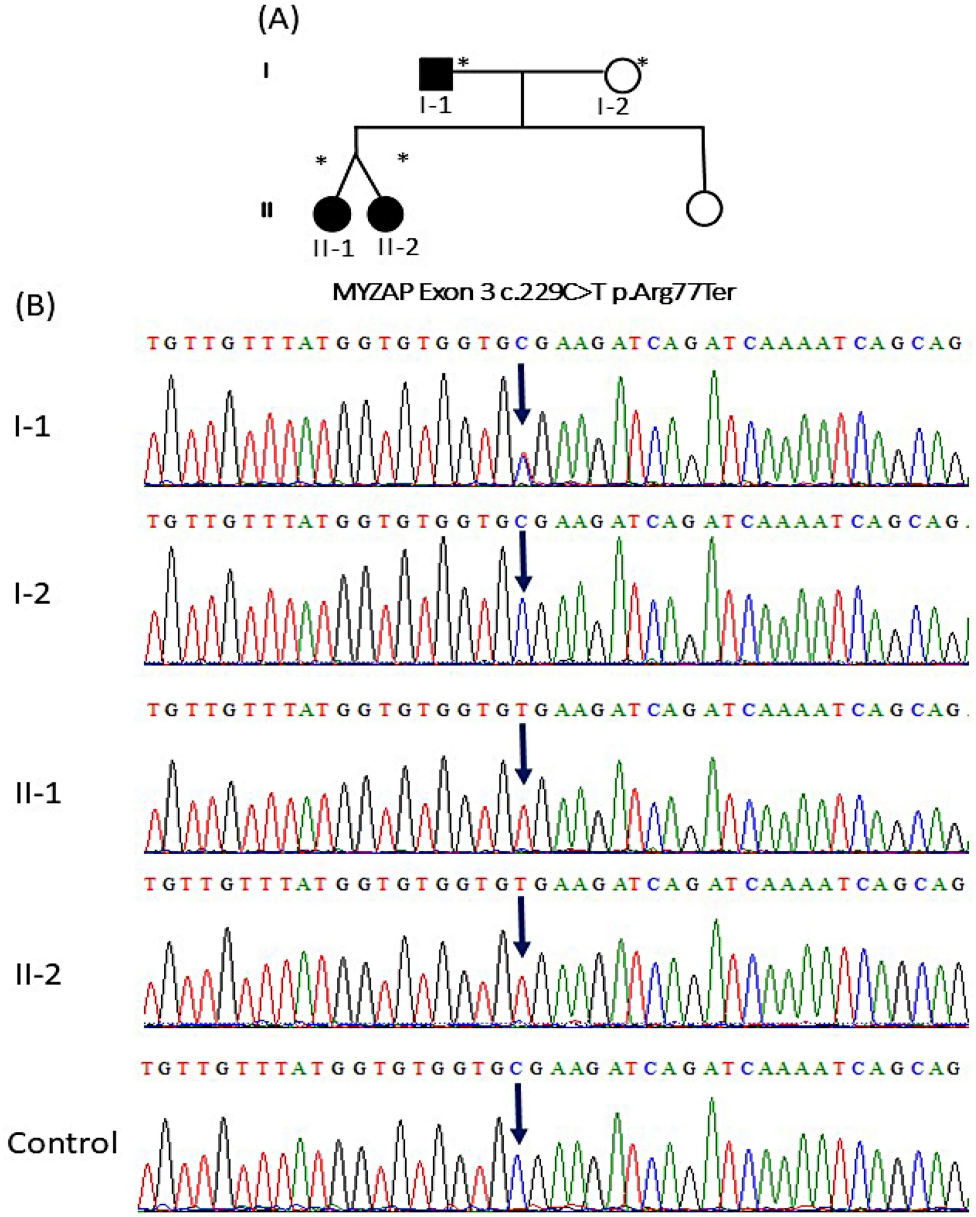
Affected family pedigree and Sanger sequencing chromatograms for MYZAP exon 3 mutation. **(A)** The family member's pedigree represents the available samples for this study. **(B)** Representative electropherogram of the *MYZAP* gene. Sanger sequencing results showing that two affected twins, II-1 and II-2, were T/T on both alleles, while the father I-1 was heterozygous (C/T), and the mother I-2 was C/C at the same position of the *MYZAP* gene.

Whole-exome sequencing identified a stop-gained variant in *MYZAP*, NM_001018100.5:c.229C > T (p.Arg77Ter), in twins II-1, II-2, as shown in the pedigree (Figure 2A), and results were validated using Sanger sequencing, as shown in Figure 2B. To the best of our knowledge, this variant has not previously been reported in association with disease or as a benign polymorphism. The p.Arg77Ter change is absent from all major population datasets except for two alleles among 111,692 individuals of non-Finnish European ancestry in gnomAD (allele frequency: 0.0018%), consistent with an extremely rare variant.

The c.229C > T substitution introduces a premature termination codon predicted to truncate the MYZAP protein. Because the stop codon occurs upstream of the region where nonsense-mediated decay (NMD) was expected, the variant was predicted to result in loss of normal gene function. Notably, a pathogenic loss-of-function variant has been reported only two residues downstream, supporting the functional importance of this region for protein stability and activity.

*MYZAP* is part of the *GRINL1A* complex transcription unit (CTU; also known as *GCOM1*), which spans the upstream *MYZAP* gene and the downstream *POLR2M* gene. Transcription from an upstream promoter generates two major transcript classes: *MYZAP-*specific (*GUP*) transcripts containing only *MYZAP* exons and *GCOM* (combined) transcripts that incorporate exons from both *MYZAP* and *POLR2M*.

**Figure 3:**
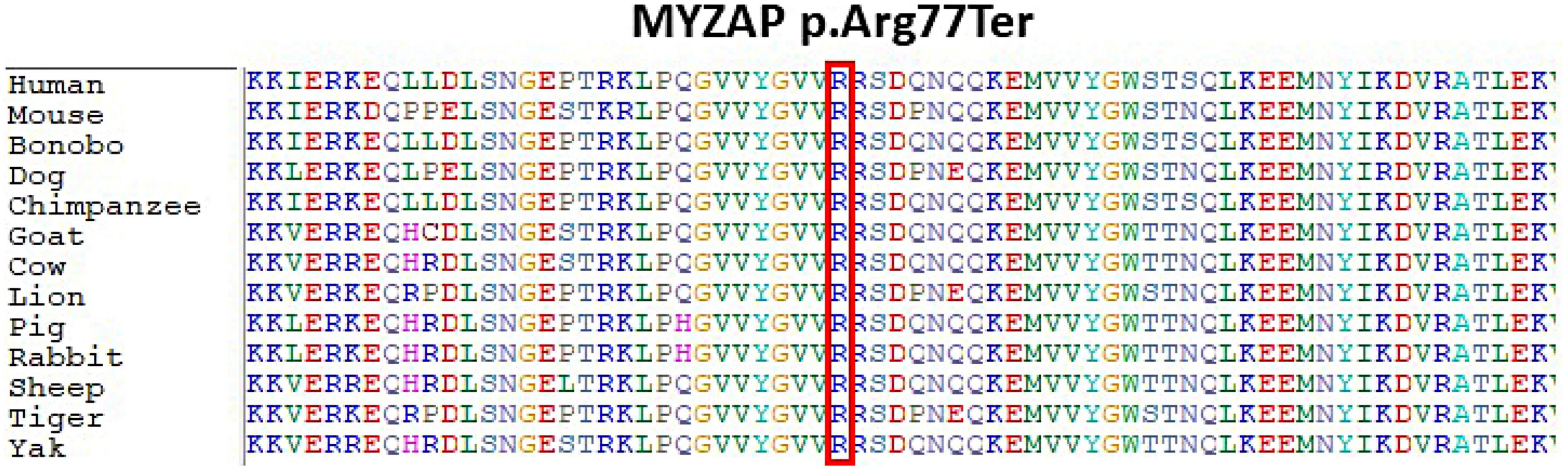
Multi-species protein sequence alignment for *MYZAP* with the p.Arg77Ter mutation. Protein alignment showed highly conserved amino acids between different species. The conserved p.Arg77Ter amino acid in MYZAP is highlighted across all species.

Variant interpretation followed ACMG/AMP 2015 standards: The p.Arg77Ter variant satisfied multiple criteria for pathogenicity, including: PVS1—Predicted loss-of-function (stop-gained) variant in a gene where LoF is a known disease mechanism. PM2—Extremely low frequency or absence in population databases. PP1—Co-segregation with disease in all affected family members. Cross-species amino acid alignment demonstrated high conservation of Arg77, further supporting the functional relevance of this site (Figure 3). Additionally, a review of ClinVar entries showed multiple pathogenic and likely pathogenic variants in *MYZAP* and *GCOM1* linked to cardiomyopathy (Table 1), reinforcing the gene-disease relationship. Furthermore, the identified *MYZAP* variant introduces a premature termination codon and is predicted to result in loss of function. The application of ACMG PVS1 evidence interpreted cautiously because *MYZAP* loss-of-function has been previously established, primarily in dilated cardiomyopathy rather than congenital cardiac septation defects. Therefore, the present findings support a possible novel association between *MYZAP* and AVSD-spectrum phenotypes but do not establish definitive disease causality.

**Table 1:** Pathogenic and likely pathogenic variants in the MYZAP and GCOM1 genes linked with cardiomyopathy reported in ClinVar so far.

Gene	DNA	Protein	Variant	Disease	References
*GCOM1, MYZAP*	c.236C > A	p.Ser79Ter	Premature truncation	Primary dilated cardiomyopathy	(9)
*GCOM1, MYZAP*	c.349C > T	p.Arg117Ter	Nonsense	Cardiomyopathy, dilated, 2K	(10)
*GCOM1, MYZAP*	c.933 + 1G > A	-	Splice donor variant	Cardiomyopathy, dilated, 2K	(10)
*GCOM1, MYZAP*	c.1150A > T	p.Lys384Ter	Nonsense	Cardiomyopathy, dilated, 2K	(11)
*GCOM1, MYZAP*	c.388C > T	p.Arg130Ter	Nonsense	Cardiomyopathy, dilated, 2K	(11)
*GCOM1, MYZAP*	c.229C > T	p.Arg77Ter	Stop gained	AVSD spectrum	Present study

The affected family members exhibited a broad spectrum of congenital cardiac developmental abnormalities, including complete AVSD with additional vascular anomalies, isolated severe mitral valve cleft/regurgitation, and partial AVSD with conduction system disease. This variable intra-familial expressivity suggests that the identified *MYZAP* variant may not act alone but rather within a complex developmental and genetic framework. Modifier genes, epigenetic mechanisms, environmental influences, and stochastic developmental variation may all contribute to the phenotypic heterogeneity observed in this family.

### 3.3 Sanger sequencing

Identified variants by WES were validated using Sanger sequencing analysis, and this confirmed a novel stop gained NM_001018100.5(*MYZAP*):c.229C > T (p.Arg77Ter) in exon 3, associated with a possible role in cardiac septation. This substitution changes a cytosine (C) to thymine (T), converting the codon for arginine (Arg) at position 77 into a premature stop codon (Ter). Arginine at position 77 is highly conserved across vertebrate species, demonstrating the functional importance of this mutation as shown in Figure 2B.

## 4 Discussion

Cardiac embryonic development is a complex biological process orchestrated by multiple genes and signaling pathways. Abnormal endocardial cushion embryonic development could lead to atrial and ventricular defects, as well as malformed atrioventricular valves (12). Several genes have been reported to be involved in the AVS pathogenesis, indicating the heterogeneity and complexity of the associated spectrum of its developmental defects.

The *MYZAP* gene encoding myocardial zonula adherens protein has been reported in several studies to play a critical structural and functional role in the intercalated discs (IDs) of the human heart, leading to dilated cardiomyopathy (13–15). However, *MYZAP's* role in congenital cardiac malformation is not yet fully understood. Here, using the whole exome sequence approach, we report for the first time a patient case with an AVSD associated with a novel stop-gained mutation NM_001018100.5(MYZAP):c.229C > T (p.Arg77Ter) in *MYZAP* exon 3. This raises the possibility that *MYZAP* could be linked to cardiac morphogenesis defects. The segregation pattern observed in this family is not fully explained by classical Mendelian inheritance, as the affected monozygotic twins were homozygous for the *MYZAP* variant, whereas the father was heterozygous and the mother appeared wild type by Sanger sequencing. Possible explanations include maternal germline mosaicism, post-zygotic mutational events, or uniparental disomy. Further studies, including SNP-array analysis, microsatellite genotyping, and parental mosaicism assessment, would be required to definitively resolve the inheritance mechanism.

Previous studies in animal models have similarly demonstrated that *MYZAP* plays an important role in both cardiac structure and/or function. In zebrafish, *MYZAP* ortholog knockdown resulted in severe contractile dysfunction (13). In mice, *MYZAP*'s expression pattern transitioned from the embryonic vasculature and endocardium (E8.0–E9.0) to a distinct cardiac pattern (E11.5–E12.5) (13). Interestingly, *MYZAP*-null (*Mzp*^−/−^) mice do not exhibit obvious cardiac morphogenesis defects at birth (16), indicating that further studies are needed to unravel *MYZAP*'s role in cardiac development fully. However, several reports have shown that a *MYZAP* mutation is linked to dilated cardiomyopathy (DCM) (9–11), atrial fibrillation (AF) (17), and biochemical stress (15, 16), indicating that *MYZAP* is important for cardiac structure and function.

Interestingly, *MYZAP*-null mice have not been reported to exhibit overt congenital cardiac morphogenesis defects, which contrasts with the phenotype observed in the present family. Several factors may account for this discrepancy, including species-specific compensatory developmental mechanisms, differences in transcript regulation, or the influence of additional genetic and epigenetic modifiers in humans. Furthermore, the identified variant may function within a broader developmental and molecular context rather than through simple haploinsufficiency alone. These observations highlight the complexity of translating animal-model findings to human congenital heart disease and emphasize the need for additional *in vitro* and *in vivo* functional studies.

It has been well-established that several transcription factors play an important role in cardiac septal formation and defects. For example, some members of TBX gene family were reported to play a key role in cardiac septation. For example, *TBX-2* was shown to contribute to the formation of the interventricular septum (18), while mutations in *TBX-1*, *TBX-5*, and *TBX-20* have been reported to be associated with VSD and ASD (19, 20). The well-known transcription factor *NKX2-5* was reported to function as a master regulator of cardiac septal development, with mutations reported to be associated with ASD and atrioventricular conduction block (21–23).

*GATA4* is an essential transcription factor that has been reported to be required for atrial and ventricular cardiac septal formation (24). Its role in septation, which was further supported by studies of the second heart field, in which reduced or absent *GATA-4* expression resulted in impaired cardiac septal formation (25). In *Gata4* mutant embryos, cardiac septation failure was rescued by *Pten*-regulated cell-cycle progression and Hedgehog signaling, confirming its key role in septation (26).

At the molecular signaling level in human cardiac septation, Hedgehog, Notch, BMP/TGF-β, Wnt/β-catenin, and FGF are known to be the main cardiac septation signaling pathways that coordinate to control septation, and impairment of these pathways can lead to atrial septal defects, VSD, ASD, and outflow tract malformations (27). *MYZAP* is primarily a structural intercalated-disc protein localized to the intercalated disc, where it contributes to the cardiomyocyte adhesion and activates Rho-dependent serum response factor (SRF) signaling. *MYZAP* effect may reflect altered cardiomyocyte adhesion sinceloss-of-function variants caused human cardiac disease, particularly cardiomyopathy (13) rather than congenital septal defects. Hence, the role of *MYZAP* in cardiac septation remains 'speculative'. In the current case report, it might be possible that *MYZAP* variants indirectly affect septum formation by influencing cardiomyocyte structural integrity. Alternatively, *MYZAP* could function as a modifier rather than acting as a master driver gene of septation, “potentially” interacting with the biologically established developmental signaling pathways involving transcription factors such as *TBX*, *NKX2-5*, or *GATA4*. Indeed, there is currently no direct experimental evidence linking *MYZAP* to these canonical septation pathways, and the possibilities mentioned here should be considered as hypotheses that require further functional *in vitro* and *in vivo* validation.

The identified *MYZAP* variant represents a strong candidate alteration associated with the AVSD spectrum observed in this family, the present study does not establish definitive causality. Functional validation studies are required to determine whether the variant directly contributes to cardiac septation defects and to clarify the underlying biological signaling mechanisms.

Although the *MYZAP* stop-gained variant represented the strongest candidate identified through WES analysis, additional rare variants were also evaluated and excluded based on population frequency, predicted pathogenicity, segregation, and phenotype correlation criteria. Nevertheless, the phenotypic heterogeneity observed within the family raises the possibility of additional modifier genes, oligogenic inheritance, or complex genetic interactions contributing to disease expression. Future studies involving larger cohorts, extended segregation analyses, and functional genomic approaches may help clarify these potential contributions.

Our study suggests that *MYZAP* could be a candidate for a congenital heart disease gene/allele, playing an important role in septation. To the best of our knowledge, this is the first report describing a possible association between atrioventricular cardiac septation defects involving a mutation in *MYZAP* gene with no evidence of cardiomyopathy. To further unravel *MYZAP's* role and function in this context, *in vitro* and *in vivo* functional studies are highly recommended to elucidate its direct and/or indirect effects on septal development. These could include gain and loss-of-function approaches to knock down and overexpress *MYZAP* in, for example, a septation-like cardiomyocyte organoid model. Also, the CRISPR/Cas9 gene-editing approach could be a feasible way to introduce patient-relevant *MYZAP* variants. The spectrum of AVSD appears to be a complex phenotype that requires further experimental investigation to be fully elucidated.

In summary, we report a rare stop-gained *MYZAP* variant identified in a family presenting with a spectrum of congenital cardiac developmental defects, including AVSD-related phenotypes. Our findings suggest a possible indirect association between *MYZAP* and cardiac septation abnormalities and support *MYZAP* as a biologically plausible candidate gene that may contribute to congenital heart disease susceptibility. However, the present study does not establish definitive causality, and further functional and genetic investigations are required to clarify the role of *MYZAP* in cardiac septation development, and disease pathogenesis.

## Data Availability

The datasets for this manuscript are not publicly available because family consents to share data publicly was not allowed. Requests to access the datasets should be directed to MN, mimrannaseer@yahoo.com.

## References

[1] Spicer RL. Cardiovascular disease in Down syndrome. Pediatr Clin North Am. (1984) 31:1331–43. 10.1016/S0031-3955(16)34725-3 6239137

[2] Belhadjer Z Pontailler M Hily M Gaudin R Raisky O Bonnet D. The particular anatomy of atrioventricular septal defect with a common valvar orifice in patients with Down syndrome: an echocardiographic study. Int J Cardiol. (2025) 423:133003. 10.1016/j.ijcard.2025.133003 39892564

[3] Samánek M Goetzová J Benesová D. Distribution of congenital heart malformations in an autopsied child population. Int J Cardiol. (1985) 8:235–50. 10.1016/0167-5273(85)90214-1 3874836

[4] Briggs LE Kakarla J Wessels A. The pathogenesis of atrial and atrioventricular septal defects with special emphasis on the role of the dorsal mesenchymal protrusion. Differentiation. (2012) 84:117–30. 10.1016/j.diff.2012.05.006 22709652 PMC3389176

[5] Becker AE Anderson RH. Atrioventricular septal defects: what’s in a name? J Thorac Cardiovasc Surg. (1982) 83:461–9. 10.1016/S0022-5223(19)37286-1 7062758

[6] Abadir S Fouilloux V Metras D Ghez O Kreitmann B Fraisse A. Isolated cleft of the mitral valve: distinctive features and surgical management. Ann Thorac Surg. (2009) 88:839–43. 10.1016/j.athoracsur.2009.06.004 19699908

[7] Jafri MA Kalamegam G Abbas M Al-Kaff M Ahmed F Bakhashab S. Deciphering the association of cytokines, chemokines, and growth factors in chondrogenic differentiation of human bone marrow mesenchymal stem cells using an *ex vivo* osteochondral culture system. Front Cell Dev Biol. (2019) 7:380. 10.3389/fcell.2019.00380 32010693 PMC6979484

[8] Pushparaj PN Abdulkareem AA Naseer MI. Identification of novel gene signatures using next-generation sequencing data from COVID-19 infection models: focus on neuro-COVID and potential therapeutics. Front Pharmacol. (2021) 12:688227. 10.3389/fphar.2021.688227 34531741 PMC8438179

[9] Maver A Zigman T Rangrez AY Coric M Homolak J Saric D. A biallelic loss-of-function variant in MYZAP is associated with a recessive form of severe dilated cardiomyopathy. Cold Spring Harb Mol Case Stud. (2022) 8:a006221. 10.1101/mcs.a006221 35840178 PMC9528970

[10] Ochoa JP Lalaguna L Mirelis JG Dominguez F Gonzalez-Lopez E Salas C. Biallelic loss of function variants in myocardial zonula adherens protein gene (MYZAP) cause a severe recessive form of dilated cardiomyopathy. Circ Heart Fail. (2024) 17:e011226. 10.1161/CIRCHEARTFAILURE.123.011226 38436102 PMC10942163

[11] Heliö K Mäyränpää MI Saarinen I Ahonen S Junnila H Tommiska J. GRINL1A complex transcription unit containing GCOM1, MYZAP, and POLR2M genes associates with fully penetrant recessive dilated cardiomyopathy. Front Genet. (2021) 12:786705. 10.3389/fgene.2021.786705 34899865 PMC8656111

[12] Person AD Klewer SE Runyan RB. Cell biology of cardiac cushion development. Int Rev Cytol. (2005) 243:287–335. 10.1016/S0074-7696(05)43005-3 15797462

[13] Seeger TS Frank D Rohr C Will R Just S Grund C. Myozap, a novel intercalated disc protein, activates serum response factor-dependent signaling and is required to maintain cardiac function in vivo. Circ Res. (2010) 106:880–90. 10.1161/CIRCRESAHA.109.213256 20093627 PMC2856095

[14] Parvari R Levitas A. The mutations associated with dilated cardiomyopathy. Biochem Res Int. (2012) 2012:639250. 10.1155/2012/639250 22830024 PMC3399391

[15] Xuan L Guo J Luo H Cui S Sun F Wang G. CCRR regulate MYZAP-PKP2-Nav1.5 signaling pathway in atrial fibrillation following myocardial infarction. iScience. (2024) 27:111102. 10.1016/j.isci.2024.111102 39507261 PMC11539591

[16] Rangrez AY Eden M Poyanmehr R Kuhn C Stiebeling K Dierck F. Myozap deficiency promotes adverse cardiac remodeling via differential regulation of mitogen-activated protein kinase/serum-response factor and β-catenin/GSK-3β protein signaling. J Biol Chem. (2016) 291:4128–43. 10.1074/jbc.M115.689620 26719331 PMC4759188

[17] Thorolfsdottir RB Sveinbjornsson G Sulem P Nielsen JB Jonsson S Halldorsson GH. Coding variants in RPL3L and MYZAP increase risk of atrial fibrillation. Commun Biol. (2018) 1:68. 10.1038/s42003-018-0068-9 30271950 PMC6123807

[18] Cervantes-Salazar JL Pérez-Hernández N Calderón-Colmenero J Rodríguez-Pérez JM González-Pacheco MG Villamil-Castañeda C. Genetic insights into congenital cardiac septal defects-a narrative review. Biology. (2024) 13:911. 10.3390/biology13110911 39596866 PMC11592229

[19] Kirk EP Sunde M Costa MW Rankin SA Wolstein O Castro ML. Mutations in cardiac T-box factor gene TBX20 are associated with diverse cardiac pathologies, including defects of septation and valvulogenesis and cardiomyopathy. Am J Hum Genet. (2007) 81:280–91. 10.1086/519530 17668378 PMC1950799

[20] Steimle JD Moskowitz IP. TBX5: a key regulator of heart development. Curr Top Dev Biol. (2017) 122:195–221. 10.1016/bs.ctdb.2016.08.008 28057264 PMC5371404

[21] Lyons I Parsons LM Hartley L Li R Andrews JE Robb L. Myogenic and morphogenetic defects in the heart tubes of murine embryos lacking the homeo box gene Nkx2-5. Genes Dev. (1995) 9:1654–66. 10.1101/gad.9.13.1654 7628699

[22] Schott JJ Benson DW Basson CT Pease W Silberbach GM Moak JP. Congenital heart disease caused by mutations in the transcription factor NKX2-5. Science. (1998) 281:108–11. 10.1126/science.281.5373.108 9651244

[23] Benson DW Silberbach GM Kavanaugh-Mchugh A Cottrill C Zhang Y Riggs S. Mutations in the cardiac transcription factor NKX2.5 affect diverse cardiac developmental pathways. J Clin Invest. (1999) 104:1567–73. 10.1172/JCI8154 10587520 PMC409866

[24] Rivera-Feliciano J Lee KH Kong SW Rajagopal S Ma Q Springer Z. Development of heart valves requires Gata4 expression in endothelial-derived cells. Development. (2006) 133:3607–18. 10.1242/dev.02519 16914500 PMC2735081

[25] Zhou L Liu J Xiang M Olson P Guzzetta A Zhang K. Gata4 potentiates second heart field proliferation and Hedgehog signaling for cardiac septation. Proc Natl Acad Sci U S A. (2017) 114:E1422–31. 10.1073/pnas.1605137114 28167794 PMC5338429

[26] Watt AJ Battle MA Li J Duncan SA. GATA4 is essential for formation of the proepicardium and regulates cardiogenesis. Proc Natl Acad Sci U S A. (2004) 101:12573–8. 10.1073/pnas.0400752101 15310850 PMC515098

[27] Lin C-J Lin C-Y Chen C-H Zhou B Chang C-P. Partitioning the heart: mechanisms of cardiac septation and valve development. Development. (2012) 139:3277–99. 10.1242/dev.063495 22912411 PMC3424040

